# Supplementary material for: Do Pictograms on Medication Packages Cause People to Consult Package Inserts Less Often? If so, With What Consequences?
Source: Behav Sci (Basel). 2023 Aug 21;13(8):696. doi: 10.3390/bs13080696 (PMC10451860; doi:10.3390/bs13080696)
Supplement: Supplementary file 1 [file behavsci-13-00696-s001.zip › behavsci-2515736-supplementary.pdf]

Supplementary Materials

Figure S1: *Example of a PI (in German)*

ANTIPAIN® lingual

Was ist ANTIPAIN® lingual und wann wird es angewendet?
ANTIPAIN lingual Tabletten enthalten den Wirkstoff Painihilatofen mit den für die wirksame Behandlung von akuten Schmerzen erforderlichen schmerzstillenden Eigenschaften.

ANTIPAIN lingual wird zur Behandlung von Schmerzen bei Kopfschmerzen, Menstruationsbeschwerden, rheumatischen Beschwerden oder leichten Zerrungen angewendet.

Darüber hinaus kann ANTIPAIN lingual bei Schmerzen nach operativen Eingriffen, Zahnschmerzen und Rückenbeschwerden eingesetzt werden.

Wie verwenden Sie ANTIPAIN® lingual?
ANTIPAIN lingual sollte vor den Mahlzeiten ohne Flüssigkeit eingenommen werden. Die Filmtabletten verlaufen auf der Zunge und können zur Halbierung oder zur Erleichterung der Einnahme geteilt werden. Trinken Sie jeweils zehn Minuten vor und nach der Einnahme des Medikaments nichts.

Wenn vom Arzt oder der Ärztin nicht anders verordnet, ist die folgende Dosierung empfohlen:

Erwachsene:

Menstruation Beschwerden, Zerrungen:
3–4mal täglich 1 Filmtablette ANTIPAIN lingual.

Kopfschmerzen/Migräne:
Bei Bedarf 1 Filmtablette ANTIPAIN lingual ohne Flüssigkeit alle 6-8 Stunden einnehmen; maximal 4 Filmtabletten ANTIPAIN lingual pro Tag.

Wann ist bei der Einnahme von ANTIPAIN® lingual Vorsicht geboten?
Während der Behandlung mit ANTIPAIN können muskuläre Spastiken, Hyperaktivität und selten Lähmungserscheinungen der äusseren Extremitäten vorkommen. Diese Komplikationen können während der Behandlung jederzeit auch ohne Warnsymptome auftreten.

Vorsicht ist angezeigt bei Patientinnen und Patienten mit einer Vorgeschichte an zentral-nervösen Erkrankungen wie Morbus Parkinson, bei Störungen der Blutgerinnung sowie bei Patientinnen und Patienten, die an Schlafstörungen, ADHS oder dissoziativen Persönlichkeitsstörungen leiden oder litten.

Dieses Arzneimittel kann die Reaktionsfähigkeit, die Fahrtüchtigkeit und Fähigkeit Werkzeuge oder Maschinen zu bedienen, massiv beeinträchtigen! Das gilt besonders für die Einnahme zusammen mit Alkohol.

Bei gleichzeitigem Alkoholkonsum können Nebenwirkungen, insbesondere, die das zentrale Nervensystem betreffen, verstärkt werden. Der Alkoholkonsum sollte während der Behandlung mit ANTIPAIN eingestellt werden.

Bei längerer Verwendung von Schmerzmitteln können Kopfschmerzen auftreten. Behandeln Sie diese nicht mit einer erhöhten Dosis des Arzneimittels, sondern informieren Sie Ihren Arzt oder Ihre Ärztin.

Darf ANTIPAIN® lingual während einer Schwangerschaft oder in der Stillzeit eingenommen werden?
Schwangerschaft:
Während der Schwangerschaft sollten sie von einer Behandlung mit ANTIPAIN absehen.

Stillzeit:
Während der Stillzeit sollten sie von Behandlung mit ANTIPAIN lingual absehen.

Welche Nebenwirkungen kann ANTIPAIN® lingual haben?
Folgende Nebenwirkungen können bei der Einnahme von ANTIPAIN auftreten. Sie sind nach ihrer Häufigkeit aufgelistet:

Häufige Nebenwirkungen die in mehr als 1 von 100 Fällen aber weniger als 1 von 10 Fällen auftreten können

- Zentralnervöse Nebenwirkungen wie Müdigkeit und Schwindel.
- Einschränkung des Reaktionsvermögens.
- Übelkeit und Erbrechen.

Gelegentliche Nebenwirkungen die in mehr als 1 von 1'000 Fällen aber weniger als 1 von 100 Fällen auftreten können

- Entzündung der Nasen- oder Rachenschleimhaut.
- Hypotonie, Synkopen.
- Muskuläre Hypertrophie, Lähmungserscheinungen in Armen und Beinen.
- Magenschmerzen.
- Überempfindlichkeitsreaktionen
- Hyperaktivität, Angstgefühle, Wahnvorstellungen im Sinne von olfaktorischen und sensorischen Halluzinationen.
- Dyspnoe, Entspannung der Muskeln der Atemwege und Atemnot, bei Patienten mit Herzinsuffizienz besteht die Gefahr eines akuten Lungenödems (Wasserlunge).

Seltene Nebenwirkungen die in mehr als 1 von 10'000 Fällen aber weniger als 1 von 1'000 Fällen auftreten können

- Angina, hohes Fieber, Anschwellen der Lymphknoten im Halsbereich.
- Muskuläre und sensorische Ausfälle, Lähmungserscheinungen an Bauch und Hals. Sollten sich Lähmungserscheinungen von den Armen langsam zu Schulter und Oberkörper ausweiten handelt es sich um einen medizinischen Notfall. Begeben sie sich umgehen in ärztliche Behandlung!
- Depressionen, Wahrnehmungsstörungen.
- Verwirrtheitszustände.
- Schläfrigkeit.
- Irreversible Sehstörung oder Sehschwäche.
- Nesselsucht, Juckreiz, Einblutung in die Haut, Schwellungen der Haut und Schleimhäute, Lichtempfindlichkeit.
- Allgemeine Schwellungen.

Table S1: *Pictogram Combinations Used*

Scenario:	Pictogram Position: 1st	2nd	3rd
Driving			
Group 1 & 2	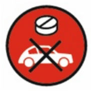	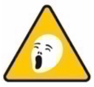	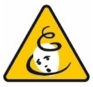
Group 3 & 4	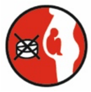	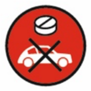	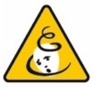
Group 5 & 6	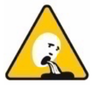	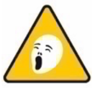	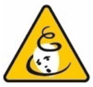
Pregnancy			
Group 4 & 5	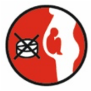	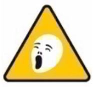	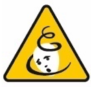
Group 1 & 6	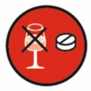	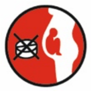	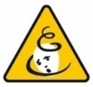
Group 2 & 3	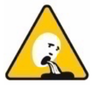	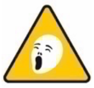	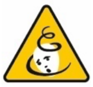
Alcohol			
Group 3 & 6	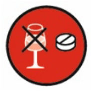	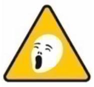	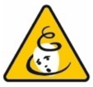
Group 2 & 5	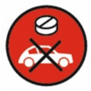	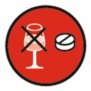	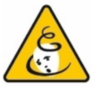
Group 1 & 4	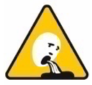	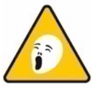	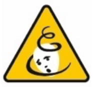

Note. The control group ("Group 7") did not see pictograms on the medication package in all three scenarios. Participants were randomly assigned to a group, and the order of presentation of the scenario per participant was also randomized.

Table S2: *Number of Participants per Factor Level Combination*

	Scenario:		
Warning:	Alcohol	Driving	Pregnancy
First	92	113	100
Second	108	98	99
None	105	94	106
Control	53	53	53
